# Preliminary Results of the Planet Nutrition Program on Obesity Parameters in Mexican Schoolchildren: Pilot Single-School Randomized Controlled Trial

**DOI:** 10.3390/ijerph18020790

**Published:** 2021-01-18

**Authors:** Diana L. Ramírez-Rivera, Teresita Martínez-Contreras, Rosa C. Villegas-Valle, Gricelda Henry-Mejia, Trinidad Quizán-Plata, Michelle M. Haby, Rolando G. Díaz-Zavala

**Affiliations:** 1 Centro de Promoción de Salud Nutricional, Departamento de Ciencias Químico Biológicas, Universidad de Sonora, Hermosillo, Sonora 83000, Mexico; a214207064@unison.mx (D.L.R.-R.); teresita.martinez@unison.mx (T.M.-C.); 2 Departamento de Ciencias Químico Biológicas, Universidad de Sonora, Hermosillo, Sonora 83000, Mexico; consuelo.villegas@unison.mx (R.C.V.-V.); trinidad.quizan@unison.mx (T.Q.-P.); haby@unimelb.edu.au (M.M.H.); 3 Departamento de Ciencias del Deporte y la Actividad Física, Universidad de Sonora, Hermosillo, Sonora 83000, Mexico; gricelda.henry@unison.mx

**Keywords:** children, obesity, prevention, school-based programs, healthy lifestyle, intervention, nutritional education, physical activity promotion

## Abstract

School-based obesity prevention programs are key to promoting healthy habits. The aim of this study was to evaluate the effect of the Planet Nutrition program on BMI z-score and other parameters compared to a control group of Mexican schoolchildren after 9 weeks of intervention. The effect of the summer holidays on the BMI z-score was also evaluated at 23 weeks. A pilot randomized controlled trial design was used and 41 schoolchildren were randomized (21 intervention group and 20 control). The program included 18 nutrition education sessions, 20 physical activity classes and six brochures for parents. At 9 weeks, no significant differences were found between the intervention and control groups in the change in BMI z-score (−0.11, 95% CI −0.23, 0.01). Significant differences were observed in some secondary outcomes: body fat percentage (−1.72, 95% CI −3.42, −0.02), waist circumference (−3.45, 95% CI −5.55, −1.36), physical activity (0.44, 95% CI 0.01, 0.88) and nutrition knowledge (1.15, 95% CI 0.27, 2.03). Summer holidays negatively affected the BMI z-score in both groups, reducing the difference observed between groups at 9 weeks (−0.07, 95% CI −0.22, 0.07). The Planet Nutrition program showed favorable effects in some obesity and lifestyle parameters in the short term.

## 1. Introduction

Childhood obesity is a global public health problem. Internationally, the prevalence of overweight and obesity in children increased from 4% in 1975 to 18% in 2016 [1]. While the prevalence has plateaued in high-income countries, it continues to increase in low- and middle-income countries [2]. In Mexico, the prevalence of overweight and obesity in schoolchildren were 35.6%, as reported by the National Health and Nutrition Survey 2018 (ENSANUT) [3].

Excess weight at early ages has physical, metabolic and psychosocial consequences [4]. Furthermore, a child with obesity is more likely to be an adult with obesity and is at increased risk of developing chronic non-communicable diseases [5]. The causes of childhood obesity are complex and multifaceted, involving genetic, biological, personal, environmental, and family behavioral factors [6]. Nowadays, children and adolescents are more exposed to obesogenic environments, which encourage excessive consumption of high energy foods and sedentary behaviors [7]. Further, rapid weight gain in children has been observed in certain periods of the year, such as the summer holidays and other festive periods [8,9,10].

The development of school-based obesity prevention programs aimed at improving nutrition and physical activity can help to promote healthy behaviors and reduce this problem [11]. A recent systematic review evaluated the effect of different obesity prevention programs that included a component of physical activity and/or nutrition. A total of 153 randomized controlled trials were included. They found that, in children aged 6 to 12 years, this type of program significantly reduced the BMI z-score compared to the control group (0.05 kg/m^2^, 95% CI 0.10, 0.01 [12]).

An important component of obesity prevention programs is nutritional education, which requires teaching materials (e.g., program handbook) to strengthen the educational process and to support implementation of the program [13]. However, few of these resources are currently available for Spanish-speaking countries. In the United States, Gortmaker et al. evaluated an obesity prevention program using a cluster randomized trial. After 2 years of intervention, a reduction in the prevalence of obesity in girls of the experimental group was observed compared to the control group. They also observed changes in some diet and lifestyle habits in both sexes [14]. With a validated intervention and handbooks available for its implementation, this program has been implemented in various schools in the United States [15].

There are few evaluations of school-based obesity prevention programs in Latin American countries, especially randomized controlled trials [16]. Additionally, few studies have produced didactic materials for the program that could potentially be used in Spanish-speaking countries. In Mexico, evaluations of programs with teaching materials are limited [17,18] and, to our knowledge, only one was conducted using a randomized controlled trial design. Thus, additional studies in the area are needed.

This research group previously worked on the development of a program currently called Planet Nutrition that includes a nutrition handbook for nutritional education sessions, in addition to physical activity classes and indirect family participation. The aim of the current study was to evaluate the effect of the Planet Nutrition program compared to a control group of Mexican schoolchildren after 9 weeks of intervention on the BMI z-score and, secondarily, on body fat percentage, waist circumference, and other physical and lifestyle variables. The study also aimed to evaluate the effect of the summer holidays on the BMI z-score of the participants at approximately 6 months from baseline.

## 2. Materials and Methods

### 2.1. Study Design

A 9-week pilot randomized controlled trial was conducted. It is a pilot study because we wanted to assess the feasibility of the program and to obtain the data to estimate a sample size for a definitive study. There were two parallel groups (intervention and control) with a 1:1 allocation ratio, stratified by sex and baseline BMI z-score. The primary outcome was the change in BMI z-score in the intervention group compared to the control group after 9 weeks. Changes in waist circumference, body fat percentage, blood pressure, cardiorespiratory fitness, self-report physical activity and sedentary lifestyle and the consumption of healthy and unhealthy foods at 9 weeks were also evaluated. Additionally, the effect of the summer holidays on children’s BMI z-score was evaluated by taking measurements at approximately 6 months from baseline (23 weeks).

### 2.2. Participants

Fifth grade students from one public elementary school in Hermosillo, Sonora, Mexico were invited to participate in the program. This school operated extended hours and the study was supported by the school authorities. The study nutrition team invited the children face to face in the classrooms to participate in March 2019. A printed invitation was delivered to the children to give to their parents, in addition to the informed consent and assent. A questionnaire was also distributed to collect personal data, including age, date of birth, history of disease, other interventions, and parents’ level of schooling. To be included in the study children had to be in fifth grade from the chosen school and be between 9 and 12 years of age. The exclusion criteria were having a medical condition at baseline or during the study, taking medication or receiving an intervention that can affect body weight, having a condition that prevents physical activity (cardiovascular, respiratory, muscular, osteoarticular, etc.) at baseline or during the study, and withdrawal of the informed consent. The intervention was conducted in the school between March and June 2019, and the 6-month measurements were conducted in early September 2019. All subjects gave their informed consent for inclusion before they participated in the study. The study was conducted in accordance with the Declaration of Helsinki, and the protocol was approved by the Research Bioethics Committee of the Department of Medicine of the University of Sonora (D-120bis). The protocol was registered retrospectively on the Clinical Trials platform (NCT04095910).

### 2.3. Training for the Study Team

Nutrition and physical activity interns from University of Sonora who implemented the intervention received a training of 10 h. For the nutrition education sessions, a nutritionist from the team trained 4 nutrition interns. They reviewed the objectives, activities and topics of the program. The physical activity training was led by an exercise specialist certified teacher from the study team. The training was given to 5 exercise specialist interns, for the planning, design and execution of the physical activity classes.

### 2.4. Intervention Components

#### 2.4.1. Nutrition Education Sessions

The study team previously worked on the development of a handbook called Planet Nutrition, which contains around 26 topics of nutrition and health (Table 1). This material was used to implement the nutrition education sessions of the program. Participants received two 1-h classes each week (18 classes in total) during regular school periods at the school library. In addition, other didactic strategies were implemented, along with the handbook, such as videos, flannel boards, sketches, games, and workshops, in order to make the classes more entertaining and comprehensive. The program centers on establishing health-related goals such as increasing consumption of fruits and vegetables, increasing physical activity time, decreasing hours spent in front of a screen, and reducing consumption of sweetened beverages. The program also includes the use of self-monitoring and positive reinforcement. Students who achieved their goals during the program were given prizes such as pencils, pens, stickers and water bottles.

#### 2.4.2. Physical Activity

The sessions were designed and implemented by the physical activity team. The classes were composed of three parts (initial, core (greater effort) and final) to improve children’s flexibility, cardiorespiratory fitness, balance, and coordination. Participants received three 1-h sessions each week (27 sessions in total) on the schoolyard court during normal school hours. These were in addition to the school’s usual physical activity classes.

#### 2.4.3. Indirect Family Participation

Six information brochures were sent to parents. These included different nutrition and health topics, such as consequences of excess weight, difference between good and bad fats, importance of physical activity, healthy eating tips, and consequences of excessive consumption of ultra-processed foods. A booklet with ideas for preparing healthy snacks for their children was also sent home to parents. The aim of involving parents was to encourage and help them to create a supportive environment for healthy behaviors outside of school.

### 2.5. Control Group

This group of children was from the same school as the participants in the intervention group. They only received general nutrition recommendations based on the 10 Tips to a Great Plate (Choose My Plate) [19], in a single session of 1 h, at the end of the study. They continued with their usual classes.

### 2.6. Outcome Measures

Measurements were conducted in the school facilities at baseline and 9 weeks. Additionally, the BMI z-score was evaluated after the summer holidays, at approximately 6 months (23 weeks). Measurements were taken by a previously trained study team.

#### 2.6.1. Primary Outcome

- BMI z-score: First, the weight and height of the children were measured. A SECA digital scale, model 872, was used to measure the body weight. The measurement was taken without shoes and accessories in the school sports uniform. Participants stood in the center of the scale with their arms at their sides. The height was measured with a stadiometer (SECA 213), without shoes, with the body resting on the stadiometer, heels together, slightly spread toes and extended legs. The Frankfurt plane was followed to a better position [20]. To obtain the BMI z-score, the WHO Anthro Plus software version 3.2 (Blue-infinity, S.A, Geneva, Switzerland) was used, where the values of weight, age, date of birth and sex were considered [21].

#### 2.6.2. Secondary Outcomes

- Waist circumference: The measurement was taken at the umbilical scar level, with the participant standing and on the upper garment (due to the lack of privacy) using a non-stretch tape measure. Participants were asked to indicate their umbilical scar location and to inhale and exhale [20].
- Body fat: A tetrapolar electrical bioimpedance device (RJL Quantum II) was used to obtain the resistance and reactance values. The equipment has an alternating electrical current of 800 µA to 500 kHz. Participants were asked to remove any metal objects, shoes, and socks. Participants lay on a mat without moving for at least 5 min. Two electrodes were placed on the wrist and two on the right-side foot. To estimate the body fat percentage, an equation validated in Mexican children was used [22].
- Blood pressure: A digital sphygmomanometer, model Omron HEM-907, was used. The children sat down and rested for 2 to 3 min, then the cuff was placed on the right arm at the level of the biceps with the arm stretched. Two measurements were made to obtain an average value of systolic and diastolic blood pressure [23].
- Cardiorespiratory fitness: The Course-Navette test was used to indirectly estimate the maximum oxygen consumption (VO_2_Max). The participants completed a 20-m shuttle run test to the rhythm of a recording. The test is in one-minute stages and the speed increases (0.85 km/h to 0.5 km/h). Children were asked to stop when they felt tired [24]. The test was carried out by the study physical activity team.
- Physical activity and sedentary activities questionnaire: The physical activity and sedentary lifestyle part of the questionnaire “The Health Behavior in School-aged Children” (HBSC) was used, which is a validated lifestyle questionnaire for school-age children [25]. It consists of 9 questions, 5 questions related to the time and frequency of physical activity and 4 to sedentary activities.
- Food frequency questionnaire: A qualitative food frequency questionnaire was used, adapted from the questionnaire used in the PERSEO program with schoolchildren [26]. The questionnaire consists of 12 questions about the consumption of healthy foods (fruits, vegetables, cereals, legumes, animal source food, dairy, etc.) and unhealthy (sugary drinks, salty snacks, sweets, pastries, sausages, etc.). The frequency ranges from never to more than twice a day [27].
- Nutrition knowledge: To assess learning in the nutrition education sessions, a questionnaire designed by the study team was used. It consisted of 32 questions about nutrition and health. Questions were multiple choice with 4 possible responses (A–D) or ‘true or false’ choices. The results were calculated on a scale from 0 to 10. The higher the score, the greater the knowledge.
- Evaluation of the acceptability and benefits of the program: At the end of the intervention, an evaluation form was completed by the children of the intervention group and their parents. The children’s evaluation contained 3 multiple choice questions to rate the program and the benefits obtained, it was evaluated at school by the program staff. Similarly, the parents’ evaluation contained 4 multiple choice questions rating their perception of benefits in their children due to the program, the kind of benefits they noticed, the rating of the information received, and the way they would like to participate in the program if offered in the future.

### 2.7. Randomization

Baseline measurements were obtained over three consecutive days. Once the measurements were completed, the children were randomly assigned to intervention or control by two persons independent from recruitment. These people were provided with the necessary data to perform the randomization (identification code, sex and BMI z-score). They used a random allocation of individuals 1:1 to the intervention group (Planet Health program) or to the control group, with randomization by blocks stratified by sex and BMI z-score. The random number sequence was generated using the randomization software, Research Randomizer^®^ (Urbaniak, G.C., & Plous, S., Lancaster Pensylvannia). There was an adequate allocation concealment of participants, because the people who conducted the allocation did it at one point in time using the database without names, just with codes. Once the randomization was completed, the nutrition team was in charge of informing the participants and their parents about the group to which they were assigned.

### 2.8. Statistical Analysis

Data were presented as means and standard deviation (mean ± SD) with 95% confidence interval. The change in the BMI z-score and the continuous variables of interest for each individual were obtained by subtracting the value obtained at the 9-week measurement from the baseline value. The BMI z-score measured after summer was obtained by subtracting the 23-week value from the baseline value. The difference between groups was obtained by subtracting the mean change in the intervention group minus that of the control. The analyses were performed using intention to treat. The missing data were replaced by the baseline value (baseline observation carried forward). An independent t-test or Mann–Whitney U-test was used, depending on the distribution of the data. For the categorical outcomes, a chi-square analysis (χ^2^) was performed for comparisons between groups. A two-tailed *p* value of <0.05 was considered as the criterion of statistical significance. NCSS version 8 (Number Cruncher Statistical System for Windows, Kaysville, UT, USA) and STATA version 14.1 (Stata Corp, College Station, TX, USA) software were used for analyses.

## 3. Results

**Figure 1:**
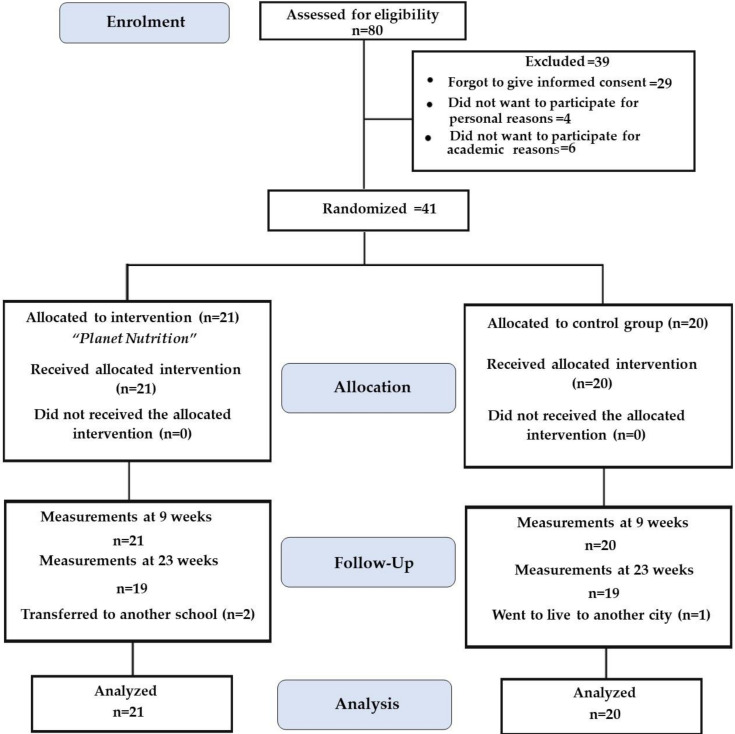
Participants flow chart at 9 and 23 weeks of the study.

All 5th grade students from the chosen school (80 students) were invited to participate in the study, of whom 39 declined to participate or did not provide informed consent on time. A total of 41 schoolchildren (21 interventions and 20 control), who gave informed consent and assent and who met the study inclusion criteria, were included (51% of the students accepted the invitation). Participants in the intervention group attended an average of 16.5 out of 18 nutrition education sessions of the program. Among the main reasons for non-attendance were school activities and illness. For the physical activity sessions, the attendance list was not taken; however, 20 sessions were implemented and students participated regularly, except in the rare event a student missed a day of school. At the end of the 9th week of intervention, there was 100% retention of the participants and 93% at 6 months (Figure 1). Baseline characteristics are shown in Table 2. Half of the participating children were males, with an average age of 10 years and a BMI z-score of 1.01 ± 0.1. No significant differences in baseline characteristics were found between both groups at the beginning of the intervention.

### 3.1. Study Variables

#### 3.1.1. Obesity Parameters

**Figure 2:**
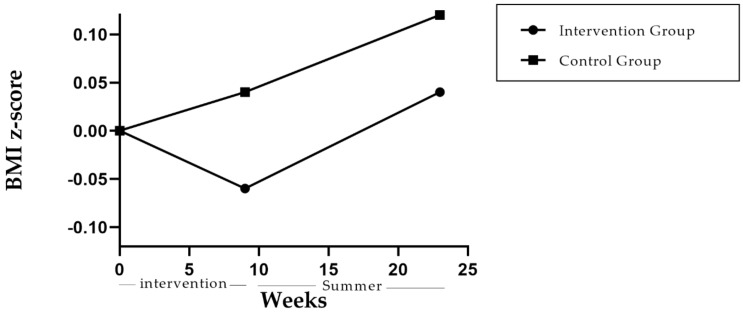
Change in BMI z-score at 9 and 23 weeks of intervention.

At the end of the 9-week program the intervention group tended towards a decrease in the BMI z-score from baseline, while the control group value increased (−0.06 ± 0.12 intervention vs. 0.04 ± 0.26 control) (Figure 2). However, the difference between groups was not significant (−0.11, 95% CI −0.23 to 0.01, SD 0.20).

For the secondary outcomes, significant differences were found between the intervention and control groups in waist circumference, body fat percentage, time of daily physical activity and nutrition knowledge (Table 3). Otherwise, there were no significant differences in systolic and diastolic blood pressure, sedentary activities and cardiorespiratory fitness.

#### 3.1.2. Lifestyle and Nutrition Knowledge

At the end of the 9-week intervention, the intervention group increased their time of daily physical activity, while the control group decreased it (0.21 ± 0.78 intervention vs. −0.23 ± 0.58 control, difference 0.44, 95% CI 0.01 to 0.88). An increase in the nutrition questionnaire score of the intervention group compared to the control group was also noted (1.67 ± 1.62 vs. 0.52 ± 1.09 control, difference 1.15, 95% CI 0.27 to 2.03). No differences were found in food consumption between groups. However, the intervention group had a tendency to improve their consumption of healthy foods (fruits and vegetables) and reduce the unhealthy ones (snacks and sugar beverages). Data about food consumption at baseline and at the end of the intervention are shown in Table A1 and Table A2.

#### 3.1.3. BMI Z-Score after the Summer Holiday

In the evaluation of the BMI z-score after the summer holiday at approximately 6 months (23 weeks), no significant differences between groups were found (−0.07, 95% CI −0.22, 0.07). However, this period affected both groups. The trend in the reduction of BMI z-score among the intervention group was lost and changed to an increase, while the control group continued with a more marked increase (0.04 ± 0.21 intervention group vs. 0.12 ± 0.26 control group at 23 weeks) (Figure 2).

#### 3.1.4. Evaluation of the Program by Children and Parents

The program was reported by children and parents to have good acceptance and benefits. The program was rated as “excellent” by 90% of the children, and 90% indicated that the intervention had benefited them somewhat. In total, 45% of children noted an improvement in eating habits, 27% mentioned acquiring more knowledge regarding nutrition and health, 23% reported performing more physical activity, and 5% reported improving their fitness. Similarly, the parents answered a survey regarding perceived changes/improvements in their children after the program. All parents (100%) indicated noticing positive changes in their children; among these, 44% reported a change in eating habits, 44% in more nutrition and health knowledge and 12% an increase of physical activity. Regarding the nutrition information that was sent to them, all parents (100%) indicated that the information was very interesting. Correspondingly, all parents reported being interested in continuing participation in this type of program, and 70% of them would like to take it online.

#### 3.1.5. Harms

No negative effect of the measurements or study activities on the health of the participants were observed.

## 4. Discussion

Despite showing only a favorable trend toward reduction of the BMI z-score, the Planet Nutrition program had a significant effect on other key variables at 9 weeks, including body fat %, waist circumference and nutrition and health knowledge, as well as in time spent in physical activity. However, no significant differences on blood pressure, diet, sedentary activities and cardiorespiratory fitness were observed. Further, the summer holiday period negatively affected the BMI z-score of both groups.

The fact that no significant difference between groups was found after the 9-week of intervention is probably explained by the limited sample size of the study (*n* = 41) and the short period of intervention. In this study, the difference in the BMI z-score between groups was −0.11, which is higher than that previously reported in a systematic review, where a difference of −0.05 between groups was found for obesity prevention programs that included a physical activity and/or nutrition component [12]. Another systematic review of school-based obesity prevention programs that included physical activity, nutrition and direct family participation, reported studies with positive effects on BMI or BMI z-score as having a standardized mean difference ranging from −0.04 to −0.27. It is worth noting that this review highlighted the importance of the program duration, where those that lasted up to 12 months showed the greatest effect on obesity parameters [28]. In the present study, despite the short intervention time (9 weeks), positive results in obesity parameters such as body fat % and waist circumference were obtained, both of which are highly relevant anthropometric indicators.

In contrast, the BMI z-score increased after the summer holidays and the favorable trend in the BMI z-score obtained with the intervention was lost but was still below that of the control group. This result is consistent with previous reports. For example, the studies by Carrel and Yin showed that schoolchildren lost the positive effect (reduction in body fat percentage and others) obtained from school-year physical activity programs, during the summer holidays [29,30]. Additionally, it has been observed that children gain more weight in summer compared to the school year, especially children with obesity [8]. This may be due to the fact that vacations days are unstructured and there is an increase in unhealthy behaviors such as increased screen time, more high-energy food consumption, irregular sleep patterns and less physical activity [31].

It is recognized that maintaining behavior change in the long term is a challenge. Behavior is the result of individual, family and community forces [32]. Given that the effect of the intervention can be lost after the intervention has finished or during the summer break [29,30], it is important to maintain the intervention throughout all the school years, not just for certain periods. Access to programs that promote healthy lifestyles during the summer (e.g., summer camps) is also needed.

The favorable results found in the Planet Nutrition program can be explained for several reasons. Firstly, the program was designed to include strategies that are associated with achieving positive changes in behavior, including motivation, risk awareness, perceived benefits of behavior change, barriers to change, skills, knowledge and behavior change strategies [33,34]. Secondly, the program includes teaching materials, with a graphic design aimed to appeal to children. Some of the sessions were adapted from other programs (previously validated by our study group) for treatment of obesity in children and adolescents [35]. Another important component was the physical activity sessions [36]. The program accumulated a total of 20 additional hours of moderate to vigorous-intensity physical activities for children, which could be a key factor in reducing body fat. For example, the study by Carrel et al. found a reduction in body fat % applying a similarly structured school program of physical activity, although for a longer period [37].

This study has some limitations. First, there was a risk of contamination between the groups because children were from the same school classrooms. However, this possible contamination would be expected to reduce the difference between groups, so the differences in the study variables could be even greater than that found. Second, the sample size was small, which limited the possibility of detecting the effect of the program on secondary outcomes. However, small samples are acceptable in pilot studies [38]. Third, the percentage of the eligible population enrolled was low (51%), which affects the generalizability of the results but not the internal validity of the study. However, participation percentages from 30 to 100% are commonly reported with these types of research studies [12].

The strengths of the study include the randomized controlled design, the excellent participation in study activities (very high attendance at nutritional education and physical activity sessions), and high participant retention.

Our study sample showed some differences in comparison to the general population of this age group. The overweight and obesity prevalences were higher than the national average in school-aged children (44% vs. 33.2%). Furthermore, despite the fact that the schoolchildren in the study were attending a public school, their parents had higher levels of schooling than the general population (55.3% had a university degree in this study vs. 9.3% national and 13% had postgraduate qualifications vs. 0.7% national) [26], so the results may not be generalizable to a national context.

The evaluation showed that the program is feasible. The program was reported by children and parents to have good acceptance and benefits, attendance at activities was high and retention was close to 100%. In previous studies, the interventionists in charge of implementing the program have usually been school teachers, which suggests a greater potential for its application in schools [39]. In our study, however, the intervention was implemented by interns of Nutrition and Physical Activity undergraduate programs who have a greater knowledge of their respective subject areas, which could be related to better results. In Mexico, it is mandatory that, after the completion of a University degree in nutrition and physical activity, interns provide social service at no cost in public institutions. Thus, in Mexico, this intervention could be implemented without cost in the long term.

## 5. Conclusions

A school-based lifestyle program that has didactic materials and includes five 1-h sessions each week (two of nutritional education and three of physical activity), implemented by nutrition and physical activity interns, has a positive impact on obesity and lifestyle parameters in Mexican schoolchildren in the short term. Despite only showing a favorable trend toward reduction of the BMI z-score, the Planet Nutrition program had a significant effect on other key variables, including body fat %, waist circumference, knowledge of nutrition and health, as well as in time doing physical activity. Additionally, adequate acceptance of the program and high retention and attendance of schoolchildren to the study activities were observed. Likewise, it was evident that the summer holiday period negatively affects the BMI z-score of both groups. Therefore, it is important to consider implementing strategies before and during the summer holidays to prevent weight gain through healthy behaviors. In Mexico and other Spanish-speaking countries, there are few studies evaluating school-based obesity prevention programs that have teaching materials to support their implementation. Therefore, this program can be a potential model to prevent the development of obesity by promoting healthy lifestyles. It is important to evaluate the effectiveness of the program on a larger scale, given that this study was only implemented in a single school.

## Figures and Tables

**Table 1:** Sessions and topics of the “Planet Nutrition” program.

Sessions	Topics
1	Creating healthy habits
2	What is excess weight?
3	Is it really bad to eat ultra-processed food?
4	The bitter truth of sweetened beverages
5	The importance of physical activity
6	Sedentary behaviors
7	Food Guidelines: My plate
8	Analyzing my healthy lunch
9	Jar for healthy drinking
10	Sweetened beverages vs. healthy lunch
11	Reading food labels
12	Importance of healthy nutrition
13	Ultra-processed food
14	Sustainable lifestyle
15	Traditional Mexican diet
16	Healthy lunch
17	Identifying good and bad fats
18	What is important to know about sodium?
19	Smoking
20	Learning about Cancer
21	Importance of consuming fruits and vegetables
22	Vitamins and minerals
23	Why is fiber consumption important
24	Gut microbiota
25	Jeopardy: Let’s put into practice the learning
26	How to prepare a salad

**Table 2:** Anthropometric, physical, lifestyle, social and family baseline characteristics of the intervention group (Planet Nutrition program) and control group participants.

Characteristics	Intervention Group (*n* = 21)	Control Group (*n* = 20)	Total (*n* = 41)	*p* Value
Mean ± SD				
Age (y)	10.2 ± 0.43	10.3 ± 0.48	10.2 ± 0.46	0.44
Weight (kg)	44.2 ± 13.5	41.8 ± 10.6	43.0 ± 12.2	0.55
Height (m)	1.44 ± 0.08	1.44 ± 0.07	2.54 ± 3.92	0.84
BMI z-score ^a^	1.14 ± 1.57	0.86 ± 1.37	1.01 ± 1.47	0.55
Waist circumference (cm)	70.6 ± 12.9	69.0 ± 11.3	69.8 ± 12.1	0.67
Body fat (%)	34.4 ± 6.52	33.5 ± 6.19	34.0 ± 6.31	0.67
Systolic Blood Pressure (mmHg)	102 ± 11.2	95.5 ± 14.9	99.0 ± 13.4	0.09
Diastolic Blood Pressure (mmHg)	60.8 ± 8.71	57.5 ± 16.3	59.2 ± 12.9	0.79
Cardiorespiratory fitness (VO_2_Max)	38.6 ± 4.09	37.9 ± 3.20	38.3 ± 1.52	0.54
Nutrition knowledge (pts) ^b^	4.80 ± 1.50	4.30 ± 1.00	4.60 ± 1.35	0.30
Daily physical activity (hrs)	1.06 ± 0.69	0.98 ± 0.47	1.02 ± 0.59	0.98
Daily sedentary activities (hrs)	0.78 ± 0.53	1.02 ± 0.63	0.90 ± 0.59	0.16
*n* (%)				
Sex				0.64
Male	10 (47.7)	11 (55.0)	21 (51.2)	
Female	11 (52.3)	9 (45.0)	20 (48.7)	
Father’s education				0.52
Basic level ^c^	5 (25.0)	2 (11.8)	7 (18.9)	
High school	4 (20.0)	7 (35.3)	11 (27.9)	
College (University)	8 (45.0)	6 (32.3)	14 (40.5)	
Postgraduate ^d^	2 (10.0)	3 (17.6)	5 (13.5)	
Mother’s education				0.09
Basic level ^c^	3 (15.8)	1 (5.26)	4 (9.75)	
High school	7 (21.1)	7 (21.1)	14 (34.1)	
College (University)	11 (63.1)	8 (47.4)	21 (51.2)	
Postgraduate ^d^	0.00	4 (26.3)	4 (9.75)	
Nutritional status (BMI-based)				0.90
Underweight	1 (4.76)	2 (10.0)	3 (7.30)	
Normal weight	9 (47.6)	11 (50.0)	20 (48.8)	
Overweight	3 (14.3)	2 (10.0)	5 (12.2)	
Obesity	8 (33.3)	5 (30.0)	13 (31.8)	

^a^ BMI z-score: body mass index, calculated as weight in kilograms divided by height in meters, expressed in units of standard deviation. ^b^ pts: points, scale 0–10. ^c^ Completion of basic level is equivalent to 9 years of schooling in Mexico. ^d^ Postgraduate refers to master’s degree or PhD.

**Table 3:** Change in obesity and lifestyle parameters of the Planet Nutrition group (*n* = 21) and control group (*n* = 20) at 9 and 23 weeks of the study.

Outcome	Baseline	9 Weeks	23 Weeks	Change at 9 Weeks ^a^	Change at 23 Weeks ^a^	Difference at 9 Weeks ^b^	Difference at 23 Weeks ^b^
Mean ± SD						Mean (95% CI)	
Weight (kg)						−0.70 (−1.35, 0.06)	−0.36 (−1.56, 0.82)
Intervention	44.1 ± 13.6	44.6 ± 13.3	46.9 ± 14.2	0.44 ± 0.78	2.75 ± 1.39		
Control	41.9 ± 10.7	43.0 ± 11.1	45.0 ± 11.7	1.15 ± 1.22	3.12 ± 2.28		
Height (cm)						0.005 (−0.0006, 0.01)	0.003 (−0.004, 0.011)
Intervention	1.44 ± 0.08	1.45 ± 0.08	1.46 ± 0.08	0.01 ± 0.01	0.02 ± 0.01		
Control	1.44 ± 0.07	1.45 ± 0.07	1.46 ± 0.07	0.01 ± 0.01	0.02 ± 0.01		
BMI z-score						−0.11 (−0.23, 0.01)	0.07 (−0.22, 0.07)
Intervention	1.14 ± 1.57	1.07 ± 1.53	1.19 ± 1.56	−0.06 ± 0.12	0.04 ± 0.21		
Control	0.86 ± 1.37	0.90 ± 1.39	0.98 ± 1.42	0.04 ± 0.25	0.12 ± 0.26		
Waist circumference (cm)						−3.45 (−5.55, −1.36) ^c^	—
Intervention	70.6 ± 12.9	68.2 ± 12.3	—	−2.35 ± 3.06	—		
Control	68.9 ± 11.3	70.0 ± 11.5	—	1.10 ± 3.55	—		
Body Fat (%)						−1.72 (−3.42, −0.02) ^d^	—
Intervention	34.3 ± 6.53	32.8 ± 6.34	—	−1.47 ± 2.76	—		
Control	33.5 ± 6.19	33.7 ± 6.62	—	0.24 ± 2.51	—		
Systolic Blood Pressure (mmHg)						−8.28 (−17.6, 1.04)	—
Intervention	102 ± 11.2	102 ± 12.7	—	−0.33 ± 11.6	—		
Control	95.4 ± 14.8	103 ± 12.9	—	7.95 ± 17.4	—		
Diastolic Blood Pressure (mmHg)						0.89 (−2.26, 4.04)	—
Intervention	60.7 ± 8.71	58.9 ± 8.7	—	−1.89 ± 2.33	—		
Control	61.7 ± 12.5	50.0 ± 13.1	—	−2.70 ± 6.72	—		
Cardiorespiratory fitness (VO_2_Max)						0.72 (−0.98, 2.4)	—
Intervention	38.5 ± 4.09	38.9 ± 4.05	—	0.35 ± 1.17	—		
Control	38.1 ± 3.13	37.7 ± 3.47	—	−0.36 ± 3.68	—		
Nutrition Knowledge (pts) ^e^						1.15 (0.27, 2.03)	—
Intervention	4.81 ± 1.56	6.49 ± 2.15	—	1.67 ± 1.62	—		
Control	4.38 ± 1.07	4.90 ± 1.27	—	0.52 ± 1.09	—		
Daily physical activity (hrs) ^f^						0.44 (0.01, 0.88)	—
Intervention	1.06 ± 0.69	1.27 ± 0.50	—	0.21 ± 0.78	—		
Control	0.98 ± 0.47	0.74 ± 0.44	—	−0.23 ± 0.58	—		
Daily sedentary activities (hrs)						0.09 (−0.29, 0.48)	—
Intervention	0.78 ± 0.52	0.78 ± 0.56	—	0.006 ± 0.74	—		
Control	1.02 ± 0.63	0.93 ± 0.68	—	−0.08 ± 0.45	—		

^a^ Obtained with the 9-week value minus the baseline value. ^b^ Defined as change for the intervention group minus change for the control group. ^c^ *p* = 0.001. ^d^ pts = points, scale (0–10). ^e^ *p* = 0.021. ^f^ *p* = 0.022.

## Data Availability

The data presented in this study are available from the corresponding author on reasonable request.

## Appendix A

**Table A1:** Baseline food consumption of the intervention group (Planet Nutrition program) and control group participants.

Characteristics	Intervention Group (*n* = 21)	Control Group (*n* = 20)	Total (*n* = 41)	*p* Value
*n* (%)				
Fruit consumption				0.43
Weekly or at least once a week	16 (76.2)	13 (65.0)	29 (70.7)	
Daily or more than 1 time a day	5 (23.8)	7 (35.0)	12 (29.3)	
Vegetable consumption				0.81
Weekly or at least once a week	14 (66.7)	14 (70)	28 (68.3)	
Daily or more than 1 time a day	7 (33.3)	6 (30.0)	13 (31.7)	
Cereals and tubers consumption				0.64
Weekly or at least once a week	9 (42.9)	10 (50.0)	19 (46.3)	
Daily or more than 1 time a day	12 (57.1)	10 (50.0)	22 (53.7)	
Legume consumption				0.47
Never or less than 1 time a week	1 (4.80)	1 (5.00)	2 (4.90)	
Weekly or at least once a week	11 (52.4)	14 (70.0)	25 (61.0)	
Daily or more than 1 time a day	9 (42.9)	5 (25.0)	14 (34.1)	
Dairy consumption				0.39
Weekly or at least once a week	12 (57.1)	14 (70.0)	26 (63.4)	
Daily or more than 1 time a day	9 (42.9)	6 (30.0)	15 (36.6)	
Animal source food consumption				0.63
Weekly or at least once a week	11 (52.4)	12 (52.2)	23 (56.1)	
Daily or more than 1 time a day	10 (47.6)	8 (40.0)	18 (43.9)	
Processed meat consumption				0.97
Never or less than 1 time a week	7 (33.3)	6 (30.0)	13 (31.7)	
Weekly or at least once a week	12 (57.1)	12 (60.0)	24 (58.5)	
Daily or more than 1 time a day	2 (9.50)	2 (10.0)	4 (9.80)	
Fat consumption				0.99
Never or less than 1 time a week	2 (9.50)	2 (10.0)	4 (9.80)	
Weekly or at least once a week	15 (71.4)	14 (70.0)	29 (70.7)	
Daily or more than 1 time a day	4 (19.0)	4 (20.0)	8 (19.5)	
Sweet beverages consumption				0.14
Never or less than 1 time a week	2 (9.5)	0 (0.00)	2 (4.9)	
Weekly or at least once a week	16 (76.2)	13 (65.0)	29 (70.7)	
Daily or more than 1 time a day	3 (14.3)	7 (35.0)	10 (24.4)	
Snack consumption				0.13
Never or less than 1 time a week	6 (28.6)	4 (20.0)	10 (24.4)	
Weekly or at least once a week	12 (57.1)	16 (80.0)	28 (68.3)	
Daily or more than 1 time a day	3 (14.3)	0 (0.00)	3 (7.30)	
Pastry consumption				0.06
Never or less than 1 time a week	5 (23.8)	1 (5.00)	6 (14.6)	
Weekly or at least once a week	14 (66.7)	19 (95.0)	33 (80.5)	
Daily or more than 1 time a day	2 (9.50)	0 (0.00)	2 (4.90)	
Candy consumption				0.33
Never or less than 1 time a week	7 (33.3)	6 (30.0)	13 (31.7)	
Weekly or at least once a week	14 (66.7)	12 (60.0)	26 (63.4)	
Daily or more than 1 time a day	0 (0.00)	2 (10.0)	2 (4.90)	

## Appendix B

**Table A2:** Food consumption of the intervention group (Planet Nutrition program) and control group participants at 9 weeks of the study.

Characteristics	Intervention Group (*n* = 21)	Control Group (*n* = 20)	Total (*n* = 41)	*p* Value
*n* (%)				
Fruit consumption				0.89
Weekly or at least once a week	12 (57.1)	11 (55.0)	23 (56.1)	
Daily or more than 1 time a day	9 (42.9)	9 (45.0)	18 (43.9)	
Vegetables consumption				0.34
Never or less than 1 time a week	0 (0.00)	1 (5.00)	1 (2.40)	
Weekly or at least once a week	11 (52.4)	13 (65.0)	24 (58.5)	
Daily or more than 1 time a day	10 (47.6)	6 (30.0)	16 (39.0)	
Cereals and tubers consumption				0.55
Weekly or at least once a week	14 (66.7)	15 (75.0)	29 (70.7)	
Daily or more than 1 time a day	7 (33.3)	5 (25.0)	12 (29.3)	
Legumes consumption				0.32
Never or less than 1 time a week	1 (4.80)	0 (0.00)	1 (2.40)	
Weekly or at least once a week	14 (66.7)	17 (85.0)	31 (75.6)	
Daily or more than 1 time a day	6 (28.6)	3 (15.0)	9 (22.0)	
Dairy consumption				0.22
Weekly or at least once a week	12 (57.1)	15 (75.0)	27 (65.9)	
Daily or more than 1 time a day	9 (42.9)	5 (25.0)	14 (34.1)	
Animal source food consumption				0.86
Weekly or at least once a week	11 (52.4)	11 (55.0)	22 (53.7)	
Daily or more than 1 time a day	10 (47.6)	9 (45.0)	19 (46.3)	
Processed meat consumption				0.73
Never or less than 1 time a week	3 (14.3)	4 (20.0)	7 (17.1)	
Weekly or at least once a week	16 (76.2)	13 (65.0)	29 (70.7)	
Daily or more than 1 time a day	2 (9.50)	3 15.0)	5 (12.2)	
Fat consumption				0.57
Never or less than 1 time a week	0 (0.00)	1 (5.00)	1 (2.40)	
Weekly or at least once a week	16 (76.2)	15 (75.0)	31 (75.6)	
Daily or more than 1 time a day	5 (23.8)	4 (20.0)	9 (22.0)	
Sweet beverages consumption				0.19
Never or less than 1 time a week	5 (23.8)	2 (10.0)	7 (17.1)	
Weekly or at least once a week	16 (76.2)	16 (80.0)	32 (78.0)	
Daily or more than 1 time a day	0 (0.00)	2 (10.0)	2 (4.90)	
Snack consumption				0.97
Never or less than 1 time a week	5 (23.8)	1 5.00)	6 (14.6)	
Weekly or at least once a week	16 (76.2)	17 (85.0)	33 (80.5)	
Daily or more than 1 time a day	0 (0.00)	2 (10.0)	2 (4.90)	
Pastries consumption				0.17
Never or less than 1 time a week	7 (33.3)	5 (25.0)	12 (29.3)	
Weekly or at least once a week	14 (66.7)	12 (60.0)	26 (63.4)	
Daily or more than 1 time a day	0 (0.00)	3 (7.30)	3 (7.30)	
Candy consumption				0.65
Never or less than 1 time a week	6 (28.6)	4 (20.0)	10 (24.4)	
Weekly or at least once a week	13 (61.9)	15 (75.0)	28 (68.3)	
Daily or more than 1 time a day	2 (9.50)	1 (5.00)	3 (7.30)	
